# Molecular epidemiology and phylogenetic analyses of human adenovirus in pediatric patients with acute respiratory infections from Hangzhou during COVID-19 pandemic

**DOI:** 10.3389/fped.2023.1237074

**Published:** 2023-08-08

**Authors:** Shuangshuang Huang, Hao Wang, Lin Li, Wenqing Xiang, Zhijian Song, Wei Li

**Affiliations:** ^1^ Department of Clinical Laboratory, Children’s Hospital, Zhejiang University School of Medicine, National Clinical Research Center for Child Health, Hangzhou, China; ^2^ Department of Bioinformatics and Computational Oncology, OrigiMed, Shanghai, China

**Keywords:** human adenovirus, epidemiology, molecular type, mutation, COVID-19

## Abstract

**Background:**

Acute Respiratory Infections (ARIs) are a major cause of morbidity and mortality worldwide. Human Adenovirus (HAdV), responsible for 5%–10% of children's ARIs, is one of the most prevalent pathogens. Our study aimed to analyze the epidemiology and phylogenesis of HAdV in pediatric patients with ARIs in Hangzhou during the COVID-19 pandemic.

**Method:**

Between November 2020 and March 2021, we collected 1,442 nasopharyngeal swabs from children with ARIs at Children's Hospital, Zhejiang University School of Medicine. Epidemiological statistics, phylogenetic and amino acid (AA) mutation analysis were conducted.

**Results:**

Our findings revealed that 386 (26.77%) samples tested positive for HAdV, with the highest rate in children aged 6–18 years and the lowest in children aged 0–1 year, indicating a different age preference of HAdV compared with pre-pandemic period. Outpatients had a significantly higher positive rate than inpatients. Moreover, patients with HAdV-coinfection exhibited more severe clinical symptoms than those with HAdV-single infection. Our phylogenetic analysis demonstrated that species HAdV-C (type 1, 2, 6) were the predominant circulating strains in Hangzhou during the COVID-19 pandemic. Further AA mutation analysis identified seventeen mutations of particular concern for biological characterization.

**Conclusion:**

In conclusion, our study provides valuable epidemiological and molecular data that will aid in epidemiological surveillance, antiviral therapies and the development of specific vaccine types, leading to improve public health.

## 1. Introduction

Worldwide, acute respiratory infections (ARIs) are a leading cause of morbidity and mortality, particularly in vulnerable populations such as children, immunocompromised cases and the elderly (1). HAdV, as one of the most prevalent pathogens, is responsible for 5%–10% of ARIs in children and 1%–7% in elderly patients (2–5).

HAdVs, first isolated from human adenoids in 1953, are nonenveloped viruses with a linear, double-stranded DNA of ∼36,000 base pairs (6). They also include nine transcription units E1–E4 and L1–L5, with the hexon gene located in L3 (7). The hexon protein, a major capsid protein with type-specific antigenic epitopes, plays a vital role in cell entry and host infection, making it crucial in the pathogenesis of HAdV infection (7, 8).

Currently, 111 different types of HAdV have been identified and classified into seven species designated HAdV A–G (http://hadvwg.gmu.edu). New types are expanding and derive primarily from homologous recombination within the same HAdV species (9). Different HAdV types cause distinct clinical symptoms due to different specific tissue tropisms (10). ARI is typically associated with species B (type 3, 7, 11, 14, 21), C (type 1, 2, 5, 6) and E (type 4) (4, 11). In addition to ARI, HAdV can also lead to other self-limiting diseases such as gastroenteritis, conjunctivitis and urinary tract infection; however, in immunocompromised patients (e.g., in oncology units and undergoing stem cell transplantation), it can cause severe complications, including hepatitis, cardiomyopathy and encephalitis, with a mortality rate up to 32.9% (10, 12–15).

Systematic reviews and meta-analyses have shown that the average prevalence rate of HAdV is 15.91% in China, 12.82% in Middle Eastern countries, and 12.58% in North African countries (16, 17). The epidemiology of HAdV is complex, with various types circulating and causing regional epidemics of respiratory infections (18). Therefore, it is essential to monitor the epidemiological features and investigate the molecular evolution process.

In this study, we focused on pediatric patients diagnosed with ARIs. ARI in children displays seasonal variation, with a significant increase during winter and spring (19). Hence, we selected the period from November 2020 to March 2021, the first period with a high incidence after the COVID-19 pandemic, to study the prevalence of HAdV in Hangzhou.

## 2. Materials and methods

### 2.1. Clinical samples

1,442 nasopharyngeal swabs were collected from pediatric patients diagnosed as ARIs at Children's Hospital, Zhejiang University School of Medicine between November 2020 and March 2021. A 2.5 ml viral transport medium80 (KaiBiLi, Hangzhou, China) was used to preserve the swabs for virus detection. The inclusion criteria were as follows: patients were diagnosed with ARIs carrying one or more respiratory symptoms such as cough, rhinorrhea, expectoration, and sore throat. Demographic data, laboratory results, and clinical symptoms of the patients were obtained from their medical records.

### 2.2. Detection of HAdV and other common respiratory viruses

Viral nucleic acids were isolated from 200 uL specimen using an EX3600 fully automatic nucleic acid extractor (Shanghai ZJ Bio-Tech Co, Ltd). Real-time PCR was used to detect HAdV, HRSV (human respiratory syncytial virus), HPIV1-3 (human parainfluenza virus1-3), HMPV (human metapneumovirus) by combining 5 uL isolated nucleic acids with 20 uL PCR reagents (Shanghai Biogerm Medical Technology Limited Company, Shanghai, China) using the Applied Biosystems 7,500 real-time PCR system (Applied Biosystems, Foster City, CA, USA). The thermocycling procedure was as follows: reverse transcription for 15 min at 50°C, denaturation for 5 min at 95°C, 45 cycles for 15 s at 95°C, and annealing for 40 s at 55 °C. The manufacturer's instructions were strictly followed during every step of the experiment.

### 2.3. Nested PCR for hexon gene of HAdV

HAdV-positive specimens were amplified by nested PCR for hypervariable region (HVR) of the hexon gene (758 bp). To start with, external PCR was conducted using F1 and R1 first-round primers. A total of 4 µl of cDNA was added to 21 µl PCR reagents (2×Taq MasterMix, CWBio Co., Ltd, China). Amplification was conducted at 94°C for 2 min, followed by 35 cycles of 94°C for 30 s, 55°C for 30 s, and 72°C for 30 s, with a final extension at 72°C for 4 min. Subsequently, nested PCR was conducted using F2 and R2s-round PCR primers with 4 µl of the external PCR products. The amplification conditions were the same as before. Finally, the PCR products were sequenced, with 93 specimens being successfully sequenced. The primers used were shown in Table 1.

**Table 1:** Primers for HAdV hexon gene amplification.

Primers	Sequences (5′-3′)
F1	GCCACCTTCTTCCCCATGGC
R1	GTAGCGTTGCCGGCCGAGAA
F2	TTCCCCATGGCCCACAACAC
R2	GCCTCGATGACGCCGCGGTG

### 2.4. Phylogenetic and AA mutation analysis

The 93 successfully sequenced nucleotides were compared to the NCBI database (http://www.ncbi.nlm.nih.gov) for preliminary genotyping. A Phylogenetic tree was constructed by the Neighbor-Joining method in MEGA-X software with a bootstrap of 1,000 replications. To perform AA mutation analysis, the first step was to compare each strain to reference strain AC_000017.1 using the BLAST software tool to identify nucleotide mutations. Then they were translated into AA using an in-house script. Finally, the amino acid mutation positions for each strain were visualized by R.

### 2.5. Statistical analysis

IBM SPSS Statistics (version: 25.0) was utilized for statistical analysis (IBM Corp., Armonk, NY, USA). The Chi-square (*χ*^2^) test and Fisher's exact test were utilized for the comparison of the categorical variable's percentage descriptions. *p*-value <0.05 was reported as statistically significant.

## 3. Results

### 3.1. The epidemiology and clinical characteristics of HAdV

Among the 1,442 pediatric patients (801 male and 641 female), 386 (26.77%) children were HAdV-positive (shown in Table 2). A slight difference in gender distribution was observed, with a higher proportion of HAdV-positive cases in male than female (29.09%, 233/801 vs. 23.87%, 153/641; *p* = 0.0261). The age of patients ranged from 2 days to 11.5 years, and the HAdV-positive rate varied significantly by age group (*χ*^2 ^= 9.942, *p* = 0.0191). Children aged 6–18 years had the highest positive rate (31.49%, 57/181), while children aged 0–1 year had the lowest (20.00%, 52/260). Of the 386 cases, only 25 were inpatients compared to 361 outpatients (13.51%, 25/185 vs. 28.72%, 361/1,257), with a significant difference being observed (*χ*^2 ^= 19.02, *p* = 0.0000).

**Table 2:** Age and sex distributions of HAdV among inpatients and outpatients.

Variable	Tested ARIs *n* (%)	HAdv-positive *n* (%)	HAdv-negative *n* (%)	Positive rate	*χ*2	*p*
Sex					4.949	**0.0261**
Male	801 (55.55)	233 (60.36)	568 (53.79)	29.09%		
Female	641 (44.45)	153 (39.64)	488 (46.21)	23.87%		
Age (years)					9.942	**0.0191**
≤1 Year	260 (18.03)	52 (13.47)	208 (19.70)	20.00%		
1–3 Years	505 (35.02)	148 (38.34)	357 (33.81)	29.31%		
3–6 Years	496 (34.40)	129 (33.42)	367 (34.75)	26.01%		
>6 Years	181 (12.55)	57 (14.77)	124 (11.74)	31.49%		
In or outpatient					19.021	**0.0000**
Inpatients	185 (12.83)	25 (6.48)	160 (15.15)	13.51%		
Outpatients	1,257 (87.17)	361 (93.52)	896 (84.85)	28.72%		
Total	1,442	386	1,056	26.77%		

Significant differences (*p* < 0.05) are shown in bold.

The 386 HAdV-positive children were diagnosed with Upper Respiratory Tract Infection (URTI) (60.62%), bronchitis (23.84%), and pneumonia (15.54%) respectively (shown in Table 3). Fever (90.93%) and cough (62.69%) were the most common symptoms. Additional clinical symptoms included expectoration (45.34%), rhinorrhea (29.53%), sore throat (4.66%), shortness of breath (3.11%), wheezing (5.96%), rales and phlegm sound (19.43%), and oxygen requirement (1.81%).

**Table 3:** Clinical characteristics of children with HAdV infection (*n* = 386).

Clinical characteristics	Number of HAdV-positive children *n* (%)
URTI	234 (60.62)
Bronchitis	92 (23.84)
Pneumonia	60 (15.54)
Fever	351 (90.93)
Cough	242 (62.69)
Expectoration	175 (45.34)
Rhinorrhea	114 (29.53)
Sore throat	18 (4.66)
Shortness of breath	12 (3.11)
Wheezing	23 (5.96)
Rales and phlegm sound	75 (19.43)
Oxygen requirement	7 (1.81)

### 3.2. Detection of viral co-infection in HAdV-positive specimens

The 386 HAdV-positive samples were analyzed for viral co-infection, 66.3% (256/386) attributed to single-infection and 33.7% (130/386) co-infection. Of the 130 co-infected children, the most prevalent virus was HRSV (70.8%, 92/130), followed by HPIV3 and HMPV.

There was a significant difference between HAdV single-infection and co-infection in age groups (*χ*^2 ^= 17.970, *p* = 0.0005). Children aged 0–3 years (including two groups ≤1Y, 1–3Y) had a higher prevalence of co-infection than single-infection, while children aged 3–18 years (including two groups 3–6Y and >6Y) had a lower prevalence of co-infection than single-infection (shown in Table 4). The co-infection group exhibited significantly higher proportions of cough, expectoration, rhinorrhea, shortness of breath, wheezing, rales and phlegm sound, and inpatients than the single-infection group (*p* < 0.05).

**Table 4:** Clinical characteristics comparison of children with HAdV single-infection and co-infection [*n* (%)].

Characteristics	Single-infection (*n* = 256)	Co-infection (*n* = 130)	*χ* ^2^	*p*
Age (years)			17.970	**0.0005** ^a^
≤1 Year	25 (9.77)	27 (20.77)		
1–3 Years	95 (37.11)	53 (40.77)		
3–6 Years	87 (33.98)	42 (32.31)		
>6 Years	49 (19.14)	8 (6.15)		
Gender (male)	155 (60.55)	78 (60.00)	0.011	0.9165^a^
Fever	235 (91.80)	116 (89.23)	0.689	0.6890^a^
Cough	124 (48.44)	118 (90.77)	71.631	**0.0000** ^a^
Expectoration	83 (32.42)	92 (70.77)	51.159	**0.0000** ^a^
Rhinorrhea	61 (23.83)	53 (40.77)	11.89	**0.0006** ^a^
Sore throat	11 (4.30)	7 (5.38)	0.229	0.6323^a^
Shortness of breath	4 (1.56)	8 (6.15)	4.606	**0.0320** ^b^
Wheezing	7 (2.73)	16 (12.31)	14.101	**0.0002** ^a^
Rales and phlegm sound	24 (9.38)	51 (39.23)	49.091	**0.0000** ^a^
Oxygen requirement	2 (0.78)	5 (3.85)	2.990	0.0840^b^
Inpatients	12 (4.69)	13 (10.00)	4.017	**0.0450** ^a^

Significant differences (*p* < 0.05) are shown in bold.

^a^ Pearson *χ*^2^ test.

^b^ Continuity correction *χ*^2^ test.

### 3.3. Phylogenetic analysis

**Figure 1:**
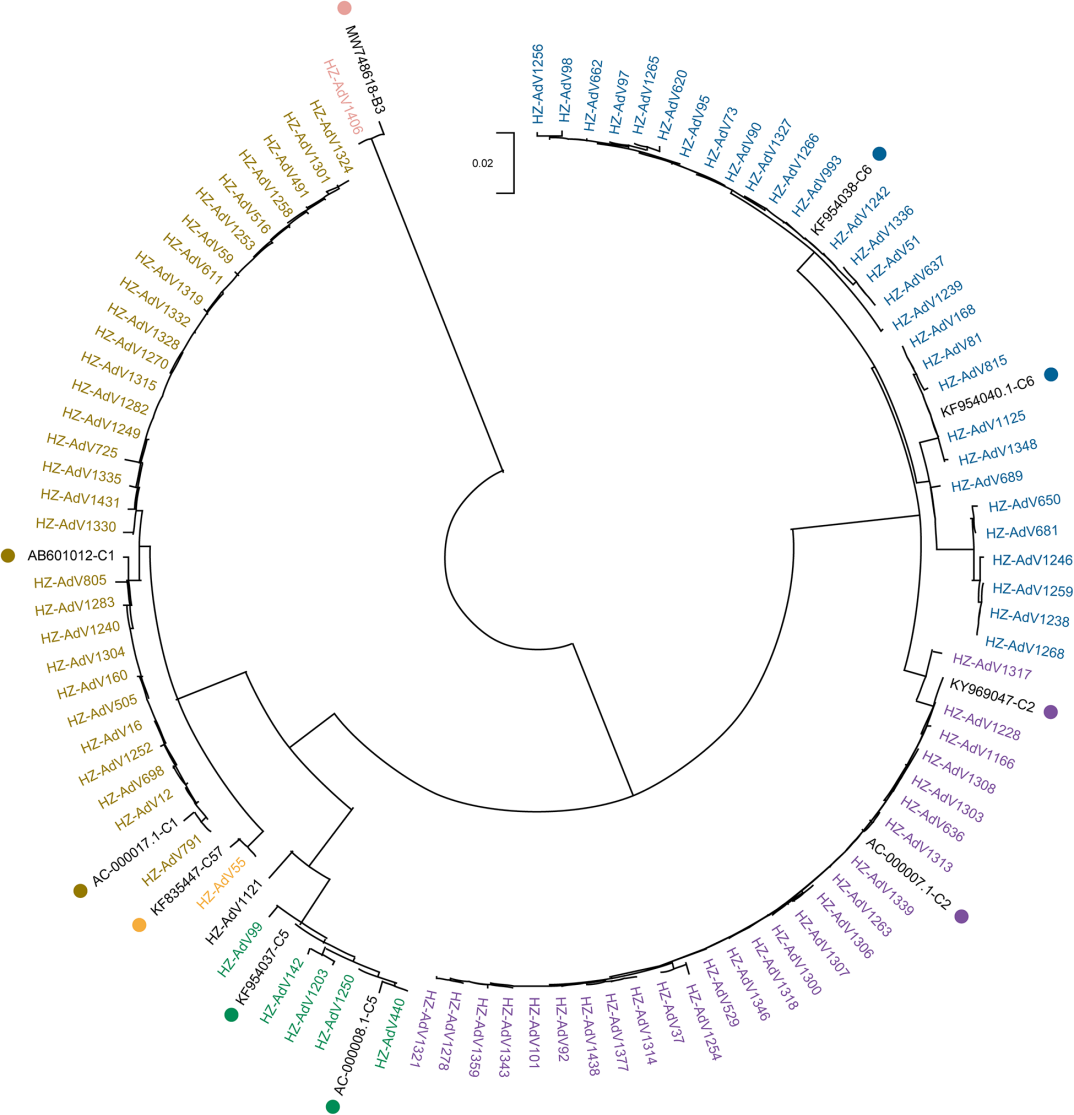
Phylogenetic tree of Hangzhou HAdV strains during November 2020–March 2021 based on hexon gene. The phylogenetic tree was constructed by the Neighbor-Joining method with a bootstrap of 1,000 replications. Reference strains from GenBank are labeled with color dots.

Phylogenetic analysis was conducted on all 93 sequenced strains and 9 reference strains obtained from NCBI. The phylogenetic tree (Figure 1) revealed that only one strain (1.08%) was HAdV-B3, which exhibited a high level of similarity to the strain isolated from Beijing in 2017 (MW748618). The remaining 92 strains (98.92%) were classified into HAdV-C, including five types: C1 (32.61%, 30/92), C2 (28.26%, 26/92), C5 (6.52%, 6/92), C6 (31.52%, 29/92) and C57 (1.09%, 1/92).

All 30 strains of the C1 type were similar to the Japanese strain (AB601012) isolated in 2010, with the nucleotide percentage concordance ranging from 98.2% to 99.7%. Likewise, the 26 C2 strains were nearly identical to KY969047 found in Thailand in 2016, with 99.2% to 100% nucleotide percentage concordance. The 6 C5 strains and 29 C6 strains were respectively comparable to KF954037 and KF954038 from Jiangsu, China in 2012. Their nucleotide percentage concordance ranged from 96.7% to 100%.

### 3.4. Amino acid mutation analysis

**Figure 2:**
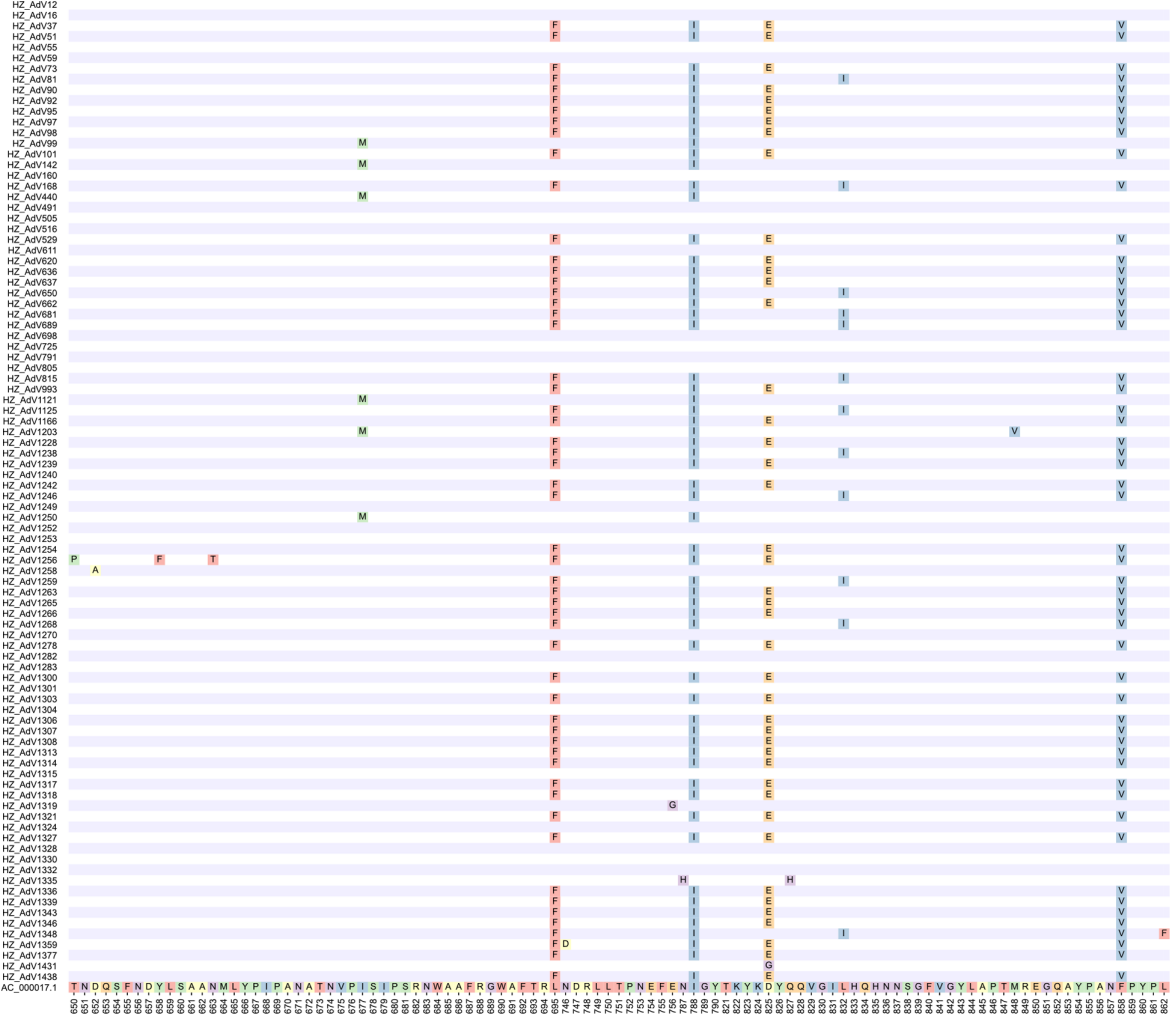
Deduced amino acid alignments of hexon gene from Hangzhou HAdV strains and reference strain AC_000017.1.

AA mutation analysis was performed on 92 strains to further investigate the molecular characteristics of HAdV-C. All mutations in different types were displayed when compared to the reference strain AC_000017.1 (Figure 2). The following AA mutations were observed: D652A, E756G, N787H, D825G and Q827H for C1 type; L695F, N746D, M788I, D825E and F858V for C2 type; I677M, M788I and M848V for C5 type; T650P, Y658F, N663T, L695F, M788I, D825E, L832I, F858V and L862F for C6 type.

Different AA mutations were observed in the same type, while the same AA mutations were shared by different types. For instance, the M788I was common among C2, C5 and C6, whereas L695F, M788I, D825E and F858V were shared by C2 and C6.

### 3.5. HAdV comparison in non-pneumonia and pneumonia children

Pneumonia is a severe respiratory infection and thus we investigated whether different HAdV types exhibited different rates of pneumonia. We analyzed the distribution of different HAdV types in non-pneumonia group and pneumonia group (Table 5). The results presented that the only B3-infected patient had pneumonia (100%, 1/1). Children infected with C5 were more likely to have pneumonia (33.3%, 2/6), followed by C2 (7.7%, 2/26) and C6 (3.4%, 1/29) (*p* = 0.004). In contrast, children infected with C1 and C57 did not develop pneumonia.

**Table 5:** Comparison of HAdV types in non-pneumonia and pneumonia groups.

Type	Non-pneumonia *n* (%)	Pneumonia *n* (%)	*p*
			**0.004**
HAdV C1	30 (100.0)	0 (0.0)	
HAdV C2	24 (92.3)	2 (7.7)	
HAdV C5	4 (66.7)	2 (33.3)	
HAdV C6	28 (96.6)	1 (3.4)	
HAdV C57	1 (100.0)	0 (0.0)	
HAdV B3	0 (0.0)	1 (100.0)	

Fisher's exact test. A significant difference (*p* < 0.05) is shown in bold.

## 4. Discussion

During the COVID-19 epidemic, various Non-Pharmacological Interventions (NPIs), such as party cancellations, closure of educational facilities and restrictions on personal movement, were implemented in China to control the spread of COVID-19 (20). Despite these measures, the positive rate of HAdV remained high. Our study revealed that between November 2020 and March 2021, 26.77% of children with ARIs in Hangzhou were HAdV-positive. This rate is higher than in previous surveys conducted in the same region (6.9%) from 2018 to 2019 (21), in Wenzhou (3.5%) from 2018 to 2019, and in Huzhou (7.08%) from 2017 to 2019 (22, 23), but lower than southern Brazil (35.8%) from 2004 to 2018 (24). These findings suggest that the prevalence rate of HAdV varies based on geographical location, sample collection time, and different study populations.

Our study revealed that the HAdV-positive rate was affected by gender and age (*p* < 0.05). Males had a slightly higher rate than females, which was inconsistent with previous studies (25, 26). This discrepancy could potentially be attributed to the fact that girls may have exhibited better adherence to NPIs such as mask-wearing and frequent handwashing. However, further investigation with a larger sample size would be required to confirm this hypothesis. Additionally, we observed that the lowest positive rate of HAdV was found in children aged 0–1 year (20.00%), whereas the highest rate occurred in children aged 6–18 years (31.49%). This finding contradicted a previous study conducted at the same hospital, which reported the highest rate in children aged 0.5–2 years from 2018 to 2019 (21). One possible explanation for this discrepancy is that during the COVID-19 pandemic, there was an emphasis on wearing masks and practicing good hand hygiene, which potentially reduced the risk of respiratory tract infections in adults and subsequently led to lower infection rates in younger children. As a result, children aged 0–1 year with maternal antibodies and limited activities, had the lowest rate. While children aged 6–18 years had the highest rate, possibly because primary and secondary schools returned to normal instruction earlier than kindergartens after the COVID-19 pandemic. These results show that the prevalence of HAdV also differs by gender and age during the COVID-19 pandemic. Therefore, continuous and unified epidemiological surveillance is urgently needed (27).

Moreover, our study found that HAdV-positive outpatients were more prevalent than inpatients (28.72%, 361/1,257 vs. 13.51%, 25/185; *p* = 0.000). This confirms the statement that most HAdV infections are mild to moderate and self-limiting, requiring only outpatient treatment (28, 29). Similar to our study, fever and cough were the most common symptoms in HAdV-positive patients (30). However, comparable clinical symptoms were found in other viral respiratory infections including HRSV, HPIV and HMPV. Therefore, the identification of pathogens is crucial for epidemiological monitoring and clinical diagnosis.

Several studies have reported co-infection of HAdV with other respiratory viruses (4, 31). Among our 130 co-infection patients, they exhibited more severe clinical symptoms than those with HAdV single-infection. Therefore, pediatricians should be aware of potential co-infection, leading to exacerbation of the disorder, increased hospitalization and more complicated therapy. As for severely symptomatic and immunocompromised patients, close monitoring of HAdV infection is required especially at an early stage due to the high mortality rate (14).

The hexon protein of HAdV comprises seven HVRs, which locate on the outer surface of the virion and contain type-specific residues responsible for viral serum neutralization (32, 33). The HVR is conserved for a given type, but varies between types, enabling type discrimination and phylogenetic analysis (34, 35). Previous studies have shown that HAdV-B (type 3, 7) were the most frequent strains in China (21–23, 25, 28, 36, 37). However, our phylogenetic tree showed that HAdV-C (type 1, 2, 6) were the predominant circulating strains in Hangzhou during the COVID-19 pandemic from November 2020 to March 2021. Previous studies have consistently associated HAdV-B (3, 7) with a higher incidence of severe respiratory disorders than HAdV-C (38–40). This change in the predominant strain from B to C during this period suggests that NPIs may have influenced the transmission of HAdV in the community, resulting in a tendency toward mild HAdV infection severity.

Furthermore, our analysis showed that C1 and C2 strains shared high nucleotide homology with AB601012 from Japan and KY969047 from Thailand, respectively. While C5 and C6 strains had high nucleotide homology with Jiangsu strains from China. These findings confirm that major strains vary geographically over time and cross provincial or national boundaries (18, 41).

Mutations are the foundation of molecular evolution. Our AA mutation results combined with the phylogenetic tree suggested that C1 and C5 were closer in the evolutionary relationship while C2 and C6 were closer among these Hangzhou HAdV-C strains. Two AA mutations (I677M and M788I) led to the splitting of C1 and C5 into two subclades, while C2 and C6 were split into another two subclades based on four AA mutations between C2 and C1 (L695F, M788I, D825E, F858V) and between C6 and C1 (L695F, M788I, D825E/L832I, F858V). Furthermore, C6 was further divided into two clusters based on one of these four mutations, namely D825E (17 strains) and L832I (12 strains). These six AA mutations between different C types confirm that the HVR is conserved for a given type but varies between types (34, 35). In addition to them, eleven mutations were also identified: D652A (AdV1258), E756G (AdV1319), N787H (AdV1335), Q827H (AdV1335), D825G (AdV1431), N746D (AdV1359), T650P (AdV1256), Y658F (AdV1256), N663T (AdV1256), M848V (AdV1203) and L862F (AdV1348). All these mutations suggest that there are differences between types during the evolution of HAdV, which may be explained by ongoing evolutionary process and possible antigenic drift. Over time, these differences may even give rise to new types (42). An in-depth exploration of these mutations may lead to a better understanding of the evolutionary mechanisms and pathogenesis of HAdV.

Adenoviral pneumonia accounts for about 10% of childhood pneumonia (43, 44). HAdV-B (type 3, 7, 21) are the most common pathogenic types of adenovirus pneumonia among children aged 0.5–5 years (45), as confirmed by the present result - the only B3-infected child was diagnosed with pneumonia. HAdV-C infections are most often subclinical or mild (46). However, the pathogenicity of pneumonia by different C types is rarely reported. Our analysis showed that children infected with C2, C5, and C6 were more likely to have pneumonia, while those infected with C1, C57 were not. This may be explained by our AA mutation results, as C1 and C57 have identical AA sequences, and compared to them, C2, C5, and C6 have 2 or 4 AA mutations, showing susceptibility to pneumonia. Whether these mutations affect the pathogenicity and virulence of viruses, larger sample sizes and more protein structure studies are needed to further confirm.

## 5. Limitation

The main limitation of our study was that it was a short-period single-center study and didn't include the various known HAdVs. To address these limitations and enhance our understanding of HAdV dynamics over time, future research should implement a long-term and multi-center surveillance approach. This would involve monitoring HAdV infections and their associated factors across different geographical locations and over an extended period. By doing so, we can obtain more comprehensive and representative data that can contribute to a broader understanding of HAdV epidemiology.

## 6. Conclusion

In conclusion, our study provides valuable epidemiological and molecular data that will aid in epidemiological surveillance, antiviral therapies and specific types of vaccine development, leading to improve public health.

## Data Availability

The datasets presented in this study can be found in online repositories. The names of the repository/repositories and accession number(s) can be found below: https://doi.org/10.6084/m9.figshare.23399495.

## References

[1] Sloots TP Whiley DM Lambert SB Nissen MD. Emerging respiratory agents: new viruses for old diseases? J Clin Virol. (2008) 42(3):233–43. 10.1016/j.jcv.2008.03.002 18406664 PMC7108325

[2] Sandkovsky U Vargas L Florescu DF. Adenovirus: current epidemiology and emerging approaches to prevention and treatment. Curr Infect Dis Rep. (2014) 16(8):416. 10.1007/s11908-014-0416-y 24908344

[3] Jain S Williams DJ Arnold SR Ampofo K Bramley AM Reed C Community-acquired pneumonia requiring hospitalization among U.S. children. N Engl J Med. (2015) 372(9):835–45. 10.1056/NEJMoa1405870 25714161 PMC4697461

[4] Xie L Zhang B Xiao N Zhang F Zhao X Liu Q Epidemiology of human adenovirus infection in children hospitalized with lower respiratory tract infections in Hunan, China. J Med Virol. (2019) 91(3):392–400. 10.1002/jmv.25333 30286268 PMC7159165

[5] Finianos M Issa R Curran MD Afif C Rajab M Irani J Etiology, seasonality, and clinical characterization of viral respiratory infections among hospitalized children in Beirut, Lebanon. J Med Virol. (2016) 88(11):1874–81. 10.1002/jmv.24544 27061822 PMC7167081

[6] Rowe WP Huebner RJ Gilmore LK Parrott RH Ward TG. Isolation of a cytopathogenic agent from human adenoids undergoing spontaneous degeneration in tissue culture. Proc Soc Exp Biol Med. (1953) 84(3):570–3. 10.3181/00379727-84-20714 13134217

[7] Shieh WJ. Human adenovirus infections in pediatric population - an update on clinico-pathologic correlation. Biomed J. (2022) 45(1):38–49. 10.1016/j.bj.2021.08.009 34506970 PMC9133246

[8] Radke JR Cook JL. Human adenovirus infections: update and consideration of mechanisms of viral persistence. Curr Opin Infect Dis. (2018) 31(3):251–6. 10.1097/QCO.0000000000000451 29601326 PMC6367924

[9] Ismail AM Cui T Dommaraju K Singh G Dehghan S Seto J Genomic analysis of a large set of currently-and historically-important human adenovirus pathogens. Emerg Microbes Infect. (2018) 7(1):10. 10.1038/s41426-017-0004-y 29410402 PMC5837155

[10] Lynch JP 3rd Kajon AE. Adenovirus: epidemiology, global spread of novel serotypes, and advances in treatment and prevention. Semin Respir Crit Care Med. (2016) 37(4):586–602. 10.1055/s-0036-1584923 27486739 PMC7171713

[11] Chen Y Liu F Wang C Zhao M Deng L Zhong J Molecular identification and epidemiological features of human adenoviruses associated with acute respiratory infections in hospitalized children in southern China, 2012–2013. PLoS One. (2016) 11(5):e0155412. 10.1371/journal.pone.0155412 27171486 PMC4865050

[12] Lynch JP 3rd Kajon AE. Adenovirus: epidemiology, global spread of novel types, and approach to treatment. Semin Respir Crit Care Med. (2021) 42(6):800–21. 10.1055/s-0041-1733802 34918322

[13] Mao NY Zhu Z Zhang Y Xu WB. Current status of human adenovirus infection in China. World J Pediatr. (2022) 18(8):533–7. 10.1007/s12519-022-00568-8 35716276 PMC9206124

[14] Peker BO Tuysuz Kintrup G Saglik I Can Sarinoglu R Guler E Mutlu D Follow-up of human adenovirus viral load in pediatric hematopoietic stem cell transplant recipients. Clin Transplant. (2021) 35(3):e14209. 10.1111/ctr.14209 33368539

[15] Kang JM Park KS Kim JM Huh HJ Ki CS Lee NY Prospective monitoring of adenovirus infection and type analysis after allogeneic hematopoietic cell transplantation: a single-center study in Korea. Transpl Infect Dis. (2018) 20(3):e12885. 10.1111/tid.12885 29569813 PMC7169713

[16] Qashqari FSI. Human mastadenovirus infections in children: a review of the current Status in the arab world in the Middle East and North Africa. Children. (2022) 9(9):1356. 10.3390/children9091356 36138665 PMC9497993

[17] Liu MC Xu Q Li TT Wang T Jiang BG Lv CL Prevalence of human infection with respiratory adenovirus in China: a systematic review and meta-analysis. PLoS Negl Trop Dis. (2023) 17(2):e0011151. 10.1371/journal.pntd.0011151 36812245 PMC9987798

[18] Mennechet FJD Paris O Ouoba AR Salazar Arenas S Sirima SB Takoudjou Dzomo GR A review of 65 years of human adenovirus seroprevalence. Expert Rev Vaccines. (2019) 18(6):597–613. 10.1080/14760584.2019.1588113 31132024

[19] Zhu Y Li W Yang B Qian R Wu F He X Epidemiological and virological characteristics of respiratory tract infections in children during COVID-19 outbreak. BMC Pediatr. (2021) 21(1):195. 10.1186/s12887-021-02654-8 33888063 PMC8060686

[20] Zhang Y Quigley A Wang Q MacIntyre CR. Non-pharmaceutical interventions during the roll out of COVID-19 vaccines. Br Med J. (2021) 375:n2314. 10.1136/bmj.n2314 34853011 PMC8634371

[21] Wang C Liu J Mi Y Chen J Bi J Chen Y. Clinical features and epidemiological analysis of respiratory human adenovirus infection in hospitalized children: a cross-sectional study in Zhejiang. Virol J. (2021) 18(1):234. 10.1186/s12985-021-01705-x 34844615 PMC8628464

[22] Xu D Chen L Wu X Ji L. Molecular typing and epidemiology profiles of human adenovirus infection among hospitalized patients with severe acute respiratory infection in Huzhou, China. PLoS One. (2022) 17(4):e0265987. 10.1371/journal.pone.0265987 35446868 PMC9022850

[23] Wen S Lin Z Zhang Y Lv F Li H Zhang X The epidemiology, molecular, and clinical of human adenoviruses in children hospitalized with acute respiratory infections. Front Microbiol. (2021) 12:629971. 10.3389/fmicb.2021.629971 33664719 PMC7921318

[24] Pscheidt VM Gregianini TS Martins LG Veiga A. Mepidemiology of human adenovirus associated with respiratory infection in southern Brazil. Rev Med Virol. (2021) 31(4):e2189. 10.1002/rmv.2189 33156553

[25] Zou L Yi L Yu J Song Y Liang L Guo Q Adenovirus infection in children hospitalized with pneumonia in Guangzhou, China. Influenza Other Respir Viruses. (2021) 15(1):27–33. 10.1111/irv.12782 32761743 PMC7767961

[26] Pan Y Zhang Y Shi W Peng X Cui S Zhang D Human parainfluenza virus infection in severe acute respiratory infection cases in Beijing, 2014–2016: a molecular epidemiological study. Influenza Other Respir Viruses. (2017) 11(6):564–8. 10.1111/irv.12514 29054112 PMC5705688

[27] Huang Y Wang C Ma F Guo Q Yao L Chen A Human adenoviruses in paediatric patients with respiratory tract infections in Beijing, China. Virol J. (2021) 18(1):191. 10.1186/s12985-021-01661-6 34556127 PMC8460180

[28] Shi J Zhou Y Wang F Wang C Miao H Sun T A case series of children with adenovirus pneumonia: three-year experiences in a tertiary PICU. BMC Pediatr. (2020) 20(1):375. 10.1186/s12887-020-02269-5 32772917 PMC7415409

[29] Edmond K Scott S Korczak V Ward C Sanderson C Theodoratou E Long term sequelae from childhood pneumonia; systematic review and meta-analysis. PLoS One. (2012) 7(2):e31239. 10.1371/journal.pone.0031239 22384005 PMC3285155

[30] Esposito S Zampiero A Bianchini S Mori A Scala A Tagliabue C Epidemiology and clinical characteristics of respiratory infections due to adenovirus in children living in Milan, Italy, during 2013 and 2014. PLoS One. (2016) 11(4):e0152375. 10.1371/journal.pone.0152375 27045588 PMC4821614

[31] Zhao MC Guo YH Qiu FZ Wang L Yang S Feng ZS Molecular and clinical characterization of human adenovirus associated with acute respiratory tract infection in hospitalized children. J Clin Virol. (2020) 123:104254. 10.1016/j.jcv.2019.104254 31901884 PMC7106522

[32] Crawford-Miksza L Schnurr DP. Analysis of 15 adenovirus hexon proteins reveals the location and structure of seven hypervariable regions containing serotype-specific residues. J Virol. (1996) 70(3):1836–44. 10.1128/JVI.70.3.1836-1844.1996 8627708 PMC190011

[33] Lukashev AN Ivanova OE Eremeeva TP Iggo RD. Evidence of frequent recombination among human adenoviruses. J Gen Virol. (2008) 89(Pt 2):380–8. 10.1099/vir.0.83057-0 18198368

[34] Kajon AE Dickson LM Murtagh P Viale D Carballal G Echavarria M. Molecular characterization of an adenovirus 3–16 intertypic recombinant isolated in Argentina from an infant hospitalized with acute respiratory infection. J Clin Microbiol. (2010) 48(4):1494–6. 10.1128/JCM.02289-09 20129962 PMC2849541

[35] Lu X Erdman DD. Molecular typing of human adenoviruses by PCR and sequencing of a partial region of the hexon gene. Arch Virol. (2006) 151(8):1587–602. 10.1007/s00705-005-0722-7 16502282

[36] Liu C Xiao Y Zhang J Ren L Li J Xie Z Adenovirus infection in children with acute lower respiratory tract infections in Beijing, China, 2007–2012. BMC Infect Dis. (2015) 15:408. 10.1186/s12879-015-1126-2 26429778 PMC4591558

[37] Wang H Zheng Y Deng J Chen X Liu P Li X. Molecular epidemiology of respiratory adenovirus detection in hospitalized children in Shenzhen, China. Int J Clin Exp Med. (2015) 8(9):15011–7. 26628985 PMC4658874

[38] Cai R Mao N Dai J Xiang X Xu J Ma Y Genetic variability of human adenovirus type 7 circulating in mainland China. PLoS One. (2020) 15(4):e0232092. 10.1371/journal.pone.0232092 32352995 PMC7192419

[39] Lu G Peng X Li R Liu Y Wu Z Wang X An outbreak of acute respiratory infection at a training base in Beijing, China due to human adenovirus type B55. BMC Infect Dis. (2020) 20(1):537. 10.1186/s12879-020-05258-2 32703176 PMC7376533

[40] Xie L Yu XF Sun Z Yang XH Huang RJ Wang J Two adenovirus serotype 3 outbreaks associated with febrile respiratory disease and pharyngoconjunctival fever in children under 15 years of age in Hangzhou, China, during 2011. J Clin Microbiol. (2012) 50(6):1879–88. 10.1128/JCM.06523-11 22442311 PMC3372113

[41] Lynch JP 3rd Fishbein M Echavarria M. Adenovirus. Semin Respir Crit Care Med. (2011) 32(4):494–511. 10.1055/s-0031-1283287 21858752

[42] Biere B Schweiger B. Human adenoviruses in respiratory infections: sequencing of the hexon hypervariable region reveals high sequence variability. J Clin Virol. (2010) 47(4):366–71. 10.1016/j.jcv.2010.01.005 20149723

[43] Chany C Lepine P Lelong M Le TV Satge P Virat J. Severe and fatal pneumonia in infants and young children associated with adenovirus infections. Am J Hyg. (1958) 67(3):367–78. 10.1093/oxfordjournals.aje.a119941 13533409

[44] Lewis PF Schmidt MA Lu X Erdman DD Campbell M Thomas A A community-based outbreak of severe respiratory illness caused by human adenovirus serotype 14. J Infect Dis. (2009) 199(10):1427–34. 10.1086/598521 19351259

[45] Huang X Yi Y Chen X Wang B Long Y Chen J Clinical characteristics of 204 children with human adenovirus type 7 pneumonia identified by whole genome sequencing in Liuzhou, China. Pediatr Infect Dis J. (2021) 40(2):91–5. 10.1097/INF.0000000000002925 33433157

[46] Garnett CT Erdman D Xu W Gooding LR. Prevalence and quantitation of species C adenovirus DNA in human mucosal lymphocytes. J Virol. (2002) 76(21):10608–16. 10.1128/jvi.76.21.10608-10616.2002 12368303 PMC136639

